# Promise or Precaution: An Exploratory Study of the Performance of OpenEvidence (OE) in Clinical Decision Making in the Global Context

**DOI:** 10.7759/cureus.118022

**Published:** 2026-10-16

**Authors:** Jennifer Benjamin, Ken Masters, Brenda Kunga, Jen-Feng Liang, Anoop Agrawal, Alvaro Margolis, Shelley Kumar, Adhari AlZaabi, Heather MacNeill, Sudha J Rajan, Sofia Valanci

**Affiliations:** 1 Pediatrics and Child Health, Baylor College of Medicine, Houston, USA; 2 Medical Informatics, Sultan Qaboos University, Muscat, OMN; 3 Critical Care, Kenyatta National Hospital, Nairobi, KEN; 4 Neurology, Taipei Veterans General Hospital, Taipei, TWN; 5 Internal Medicine, Baylor College of Medicine, Houston, USA; 6 Continuing Professional Development, RedEMC, Montevideo, URY; 7 Pediatrics, Texas Children's Hospital, Houston, USA; 8 Human and Clinical Anatomy, Sultan Qaboos University, Muscat, OMN; 9 Medicine, Sinai Health System, University of Toronto, Toronto, CAN; 10 Internal Medicine, Christian Medical College, Vellore, IND; 11 Internal Medicine, Cleveland Clinic Lerner College of Medicine, Cleveland, USA

**Keywords:** artificial intelligence, clinical decision making, evidence based medicine, global use of ai, openevidence

## Abstract

Background

Clinicians face the challenging task of keeping up with evidence-based literature to inform clinical decision-making. OpenEvidence (OE) (OpenEvidence, Miami, USA) holds great promise by providing the latest evidence to inform patient care management globally.

Objective

To explore the performance of OE in a global context.

Methods

Eleven clinicians across six countries, including the United States, Canada, Uruguay, Kenya, Taiwan, and Oman, performed standardized searches for 10 common clinical conditions, with each query identifying the clinician’s institution and location. OE search outputs (80 evaluations) were evaluated using the Rubric Assessment Scale (RAS). Additional questions compared US-based results versus other countries. Data were analyzed using descriptive statistics. Differences between US based results and the global group were assessed using the Wilcoxon rank-sum test to compare median values as part of an exploratory proof of concept study.

Results

OE performed well on core quality metrics. About 88% of the searches were rated as “completely” or “almost entirely” accurate, and 89% were “efficient and succinct” or “mostly efficient,” as rated by the authors, with lower scores of 73% for comprehensiveness, 75% for argument and evidence, and 65% for creativity and originality.

US-based searches demonstrated alignment with management guidelines (p=0.02), with higher perceived overall value (p=0.02) and more frequent inclusion of locally authored references by experts (p<0.0001). Greater variability in differential diagnosis was observed in the global group [interquartile ranges (IQR) 1-5 vs 1-2: p=0.03], reflecting wider rater disagreement rather than less relevant differentials, alongside a notable lack of region-specific management recommendations. Of the search results, most references originated in the US, with no Global South references, lacking contextualized management based on regional society guidelines. OE results lacked epidemiologically relevant differentials such as malaria for a febrile child in Kenya. Accessibility to OE was variable, with India lacking access and several Global South countries experiencing variable access.

Conclusion

In this exploratory study, OE returned an evidence base that was overwhelmingly US-derived, lacking Global South references, and did not vary with geographic origin of query even when the location was explicitly requested. There was output variability in management suggestions, with US-based searches showing greater alignment with clinical guidelines and locally authored references compared to global searches. These findings are hypothesis-generating and cannot establish causal mechanisms; given the exploratory design and small sample size with convenience sampling, the results should be interpreted cautiously. Clinicians need to critically analyze and evaluate artificial intelligence (AI) outputs and be aware of inherent biases before applying them to clinical practice, especially when caring for patients in global contexts.

## Introduction

Clinicians are faced with a rapid acceleration of knowledge dissemination, making it increasingly difficult to stay current; most health professionals are inundated with the exponential increase in the amount of scientific literature [1,2]. Traditional point-of-care tools such as DynaMed and UpToDate provide curated summaries but require subscription access and structured navigation rather than conversational natural language queries [3]. Recent advances in large language models (LLMs) have introduced a new class of artificial intelligence (AI)-enabled clinical decision support tools capable of responding to natural language queries. Evaluation of general-purpose and medically trained LLMs has demonstrated promising performance on standardized medical examinations and structured case-based reasoning tasks [4-6]. Although LLMs are increasingly used in health care contexts, there is a need for rigorous output evaluation [7] due to concerns with hallucinations, overconfidence, limited transparency in processing, and susceptibility to medical misinformation [8].

OpenEvidence (OE) is an open-access proprietary AI-powered LLM trained on peer-reviewed biomedical literature and designed to support clinicians in real-time decision-making [9]. Unlike traditional databases, OE allows natural language queries and provides synthesized responses with cited references. It has additional functionalities, such as the ability to perform deep searches on challenging topics, suggest workup of complex patients, draft patient prior authorization letters, generate patient education materials, perform calculations (e.g., ionized calcium levels), and provide continuing medical education (CME) credit activities. As an open-access tool, OE has been positioned as a means of democratizing access to medical knowledge globally [10].

In theory, AI systems trained on sufficiently diverse and representative datasets could be adapted to reflect local epidemiology, sociocultural factors, and health systems [11]. However, empirical evidence demonstrating effective geographic personalization in LLMs remains limited [6,12,13]. In addition, AI solutions hold immense transformative potential for providing accurate, context-specific, objective, and evidence-based suggestions for clinical decision-making [3,4,14]. Generative AI (GenAI) tools, like OE, require pre-training datasets and retrieval-augmented generation (RAG) to improve the accuracy of outputs in response to user prompts. The output of AI models is influenced by the algorithms that assign values and biases embedded during their development, using parameters such as diagnosis, medication choices, and management options directly relevant to patient care [15].

Therefore, if the pre-training dataset predominantly consists of literature from a specific country or region without the ability to generalize, these tools can suggest incorrect recommendations when used outside those contexts. In the absence of contextual pretraining data, these tools can exacerbate healthcare disparities instead of addressing them [16]. For example, immunization schedules vary among different countries, lacking generalizability, and the management of clinical conditions needs to consider regional guidelines for patient care [17-19].

Given the rapid integration of GenAI tools into clinical workflows and the lack of empirical data evaluating their performance across diverse geographical settings, we sought to assess whether OE response outputs vary based on the users’ global context and location. Specifically, we aimed to evaluate the overall quality of OE responses using a validated evaluation instrument. The rubric assessment scale (RAS) [20] assesses the degree to which recommendations align with region-specific guidelines and sociocultural considerations. We hypothesized that GenAI tools such as OE have the potential to democratize access to scientific literature and provide region-specific management advice grounded in local guidelines that reflects geographic variability. We hypothesized that GenAI tools like OE have the potential to democratize access to scientific literature and provide region-specific medical management advice grounded in local guidelines incorporating geographic variability. We acknowledge, however, that it cannot be confirmed whether OE’s algorithm addressed the geographic context as intended with the prompting.

Recent evaluations have demonstrated contrasting conclusions about OE’s performance relative to frontier general purpose LLMs. Vishwanath et al. found that frontier models outperformed OE across all evaluation stages in queries that were designed in a single US setting, with OE scoring lowest in communication clarity and incomplete clinical content [21]. Contrasting these findings, another study rated OE responses as more accurate, clinically useful, better sourced, and easily verified, concluding its performance was better than frontier models [22]. Both of these recent studies were performed with US-based queries without comparing US-based queries with global contexts where guidelines and disease epidemiology differ substantially; our study addresses that gap with an exploratory proof-of-concept study. A previous version of this article was posted to the medRxiv preprint server on October 30th, 2025.

The objectives of this exploratory study were to: a) evaluate the quality of OE responses to clinical queries, as rated by clinicians using the RAS. b) compare OE responses to queries from the United States and from other countries for guideline alignment with regional standards. c) evaluate contextual adaptation of OE responses, including incorporation of region-specific epidemiology and management into clinical queries; and d) appraise the citations and references and their sources cited as part of OE responses.

## Materials and methods

Study design

We examined the performance of OE recommendations across diverse geographic contexts using a cross-sectional design. For our process, we followed the Chatbot Assessment Reporting Tool (CHART) guidelines (Appendix 1 ) [23]. We used a proprietary, medical-domain-specialized LLM, accessible to clinicians with national provider numbers. We recruited a convenience sample of 12 clinicians from diverse specialties. Authors’ geographic locations were distributed across several countries, including the United States (n=3), Canada (n=1), Kenya (n=1), Uruguay (n=1), Taiwan (n=4), Oman (n=1), and India (n=1). For evaluating search outputs, our inclusion criteria included only clinicians who (1) held an active national provider number permitting access to OE, (2) practiced in a defined geographic region, (3) were able to evaluate at least one clinical scenario within their specialty, and 4) conducted OE searches between June and July 2025. Clinicians were excluded if (1) OE access could not be established, (2) their practice could not be assigned to a single geographic region, or (3) they did not complete any scenario evaluation within the June-July 2025 window. The geographic origin of authors was determined through a convenience sample of authors and collaborators available for this exploratory study. Of 12 clinicians recruited, one (India) was excluded, as OE access could not be established with an Indian national provider number and hence no searches were possible, yielding a final sample of 11 raters across six countries. At the query level, clinicians were restricted to clinical scenarios within their specialty or scope of practice, to ensure ratings of OE output reflected informed clinical judgement rather than general impressions of the response and to prevent conflation of poor specialty-specific knowledge with substandard responses. We used the basic LLM functionality of OE without any additional features.

Ethical considerations

We did not obtain IRB approval as the search queries were performed by authors on hypothetical clinical queries not involving real patients or identifiable data. In addition, this was a proof-of-concept exploratory research study and had no more than minimal risk. Participation was voluntary; anonymity and confidentiality were ensured. The OE management suggestions were only used for critical appraisal and analysis without impacting patient care management. The primary reason for the research was to critically analyze and appraise results from OE search queries, which are a required competency for clinicians.

Data collection and query development

A standardized set of 10 natural language hypothetical clinical queries representing common and prevalent conditions (e.g., childhood asthma, group A streptococcus infection, hypertension, pneumonia) was developed (Appendix 2). The clinical prompts were developed collaboratively by the study team. Each clinician investigator contributed real-world cases of common clinical conditions whose diagnosis and management vary geographically.

Prompts were written in natural language to mirror how clinicians query decision support tools in practice and covered several clinical tasks, including management, treatment planning, and differential diagnosis. This approach allowed us to assess whether OE's responses differed across settings for both diagnostic, management, and differential diagnosis questions. The first and senior authors edited the prompts for clarity, and subspecialty clinicians verified their clinical accuracy. Each prompt was constructed to include explicit geographic context. Each investigator began the prompt with their institutional affiliation, clinical role, specialty, and country, followed by the standardized clinical scenario and question. Some queries included follow-up questions prompting OE to demonstrate contextual adaptation of management suggestions with adherence to local guidelines. This was intended to additionally elicit OE responses specific to the geographic query location, so that observed differences in output would reflect the tool's responsiveness to contextual adaptation to real-world clinical queries. Each prompt was entered in a separate chat session, and the date and location of each search were recorded. All responses were saved to a shared document. To ensure relevant and accurate evaluation, clinicians assessed only responses pertinent to their own clinical specialty and geographic region. Patients and the public were not involved in prompt development. Search results with date and location were noted for each prompt. See Appendix 3 for all prompts and their corresponding responses from each location.

The prompt was input into separate chat sessions, and all the responses were saved and uploaded into a shared document.11 clinicians used the RAS to evaluate the search results from 10 clinical case queries. The primary evaluation of the search results was conducted using a previously validated instrument for assessment of large language model responses: the RAS (Table 1) [20], with additional questions regarding differential diagnoses and clinical management (Table 2). The RAS was designed for AI-generated outputs with formal reliability testing and factor analysis evaluating quality of content. Its structured criteria and defined scoring levels provide a standardized framework supporting consistency across outputs generated in varied settings. Grammar and spelling responses scored 1 (excellent), with zero variance, and the dimension is therefore not reported as part of the primary analysis. We also explored OE's ability to provide region-specific differential diagnoses and summarized search results using group consensus.

**Table 1:** Rubric assessment scale Reproduced from [20].

Relevance	Fully and directly addresses all aspects of the task.	Directly addresses most aspects of the task; minor points missing.	Partially addresses the task but lacks important details.	Vaguely related but does not directly address the task.	Unrelated to the task.
Accuracy	Completely accurate.	Almost entirely accurate.	Mostly accurate but contains minor inaccuracies.	Contains a few factual inaccuracies.	Contains several factual inaccuracies.
Efficiency	Efficient and succinct.	Mostly efficient, with minor room for improvement.	Somewhat efficient, with room for improvement.	Could be more succinct or well-structured.	Unnecessarily lengthy or convoluted.
Coherence	Completely coherent and easy to follow.	Very coherent with only minor issues.	Mostly coherent, with a few confusing statements.	Some coherence but difficult to understand.	Largely incoherent or nonsensical.
Comprehensiveness	Extremely comprehensive, covering all aspects of the task in detail.	Quite comprehensive, only missing a few minor points.	Covers most of the task but lacks some details.	Covers a few elements of the task but misses many key points.	Barely touches on the task.
Grammar and Spelling	Excellent grammar and spelling with no errors.	Good grammar and spelling with a few minor errors.	Acceptable grammar and spelling, but with several mistakes.	Poor grammar and spelling with many errors.	Unacceptable grammar and spelling with frequent errors.
Argument and Evidence	Clearly articulated, strong argument supported by substantial evidence.	Solid argument with adequate evidence.	Argument present but lacks substantial supporting evidence.	Weak argument with little or insufficient evidence.	Absent or unclear argument with no evidence.
Language and Tone	Highly sophisticated and nuanced language and tone.	Sophisticated language and tone with minor inconsistencies.	Acceptable language and tone but with some inconsistencies.	Inappropriate or inconsistent language and tone.	Poor language use and inappropriate tone.
Creativity and Originality	Unique, insightful, and innovative approach.	Somewhat unique with some insightful thoughts.	Ordinary approach with few insights.	Lack of originality, few insights.	Completely lacks creativity or originality.

**Table 2:** OE specific questions to evaluate output and responses Additional questions specific to OE were created by the authors through group consensus. OE: OpenEvidence.

Additional items (Likert 1 = Strongly agree, 2 = Agree, 3 = Neutral, 4 = Disagree, 5 = Strongly Disagree)					
The management plans were biased					
The management plans were current and aligned with guidelines					
The diagnosis suggested aligns with the primary diagnosis					
The differential diagnosis listed by OE aligns with my top 5 differential diagnoses					
OE identified conditions I may have missed on my own					
OE suggested differential diagnoses that were irrelevant					
The references listed were appropriate to the case					
The references included articles authored by local experts/ local institutions					
Use of OE-generated management by an inexperienced provider may cause harm					
Overall, OE was valuable					

Statistical approach

Statistical Analysis was conducted in two parts. First, we assessed the overall performance across search results by summarizing the RAS ratings using descriptive statistics [medians with interquartile ranges (IQR) and supplemented with means with standard deviations (SD)]. We reverse-coded RAS criteria to align with additional questions about differential diagnosis and clinical management queries. Second, we conducted a comparative analysis between searches conducted by clinicians based in the US and in global locations. The Wilcoxon rank-sum test, a nonparametric test appropriate for ordinal data and small samples that does not assume a normal distribution, was used to analyze the median difference in scores on the additional questions between these two groups. All quantitative data were analyzed using SAS version 9.4 (SAS Institute Inc., Cary, USA). A total of 80 RAS ratings were collected across the 10 clinical cases by 11 international clinician raters. Not all raters assessed all cases, as evaluations were limited to a clinician's area of expertise. Given the small sample and the exploratory design, no a priori power calculation was performed, and all statistical comparisons are considered hypothesis-generating.

## Results

Overall performance on rubric assessment scale

OE performed well on core metrics of search quality. Across all cases, raters scored search results favorably: search results rated 88% for accuracy as “completely or entirely accurate”, and 89% rated efficiency as “efficient and succinct” or “mostly efficient”; 87.5% rated coherence as “very coherent with minor issues or completely coherent”, and 85% rated language and tone as “sophisticated and highly sophisticated”. Raters gave lower scores in areas requiring deeper clinical and contextual nuance. Comprehensiveness and argument and evidence ranked 73% and 75% for the top two categories on RAS, respectively. Raters noted that the search results often lacked contextual adaptation, such as decision aids for admission versus discharge, and did not consistently integrate regional guidelines and socio-cultural contexts with authoritative, evidence-based recommendations. The lowest-scoring domain was Creativity and Originality (65%), reflecting raters' assessments that OE's differential diagnoses lacked incorporation of region-specific epidemiology. A summary of RAS rating analysis is provided in Table 3.

**Table 3:** Rubric assessment scale scoring of OE clinical queries 1 = best rating; 5 = worst rating OE: OpenEvidence. SD: standard deviation.

	Median (IQR)	Mean	Mean range
		(SD)	Across all cases
			(Lowest- highest)
Relevance	1 (1-3)	1.8 (0.96)	1.25-2.25
Accuracy	1 (1-2)	1.6 (0.71)	1.29-1.83
Efficiency	1 (1-2)	1.6 (0.76)	1.33-1.80
Coherence	1 (1-2)	1.5 (0.70)	1.25-1.60
Comprehensiveness	1.5 (1-2.5)	1.9 (1.07)	1.43-2.33
Argument and Evidence	1 (1-2)	1.7 (0.92)	1.50-2.29
Language and Tone	1 (1-2)	1.6 (0.77)	1.50-1.89
Creativity and Originality	2 (1-3)	2.2 (1.1)	1.67-2.50

Comparison of searches based in US vs. global locations

We conducted a comparative analysis of responses from US-based clinicians (n=3, 21 ratings) versus the searches conducted in various global locations (n=8, 59 ratings) using a series of additional questions (Table 4 ). All comparative analyses are exploratory given the sample imbalance (n=21 US ratings Vs 59 global ratings). Both groups found that OE's primary diagnosis aligned with their own [median (IQR) 1 (1-2) US vs global 2 (1-2); p=0.11], and a significant difference was observed in the assessment of the differential diagnoses. The searches conducted in global locations reported wider variability in the relevance of the suggested differential diagnosis [median (IQR) 2 (1-2) vs 2 (1-5); p=0.03], with a lack of region-specific management recommendations.

**Table 4:** Comparison of OE outputs between US and other countries Statistical significance was evaluated using the two-sample Wilcoxon rank-sum test (reported via SAS Proc Npar1way as the rank-sum statistic, S, along with its standardized Z-score approximation). OE: OpenEvidence.

Items	Median		S-Statistic	Z score	p
	(Interquartile range)				
	USA	Others (n=59)			
	(n=21)				
Management plans were biased	4 (5-5)	4 (4-5)	544	1.4	0.163
Management plans aligned with guidelines	1 (1.5-2)	2 (2-2)	365	-2.39	0.017
Diagnosis aligned with primary diagnosis	1 (1-2)	1 (2-2)	717	-1.62	0.104
Differential aligned with my top 5	1 (2-2)	1 (2-5)	653	-2.23	0.026
OE identified conditions I may have missed	3 (4-4)	2 (4-5)	871	0.23	0.821
OE suggested irrelevant differential diagnoses	4 (4-5)	4 (5-5)	740	-1.3	0.193
References were appropriate to the case	1 (2-2)	1 (2-2)	783	-0.8	0.423
References included local experts/institutions	1 (2-2)	4 (5-5)	300	-6,27	<0.001
Management by inexperienced provider may harm	2 (3-4)	2 (3-4)	896	0.64	0.524
Overall, OE was valuable	1 (1-2)	2 (2-2)	576	-3.22	0.001

Three US-based raters found the management plans to be better aligned with established guidelines compared to the raters in the global group [median (IQR), 1.5 (1-2) vs 2 (2-2); p=0.02]. This corresponded with a higher perceived overall value of OE among the US raters [median (IQR), 1 (1-2) vs 2 (2-2); p=0.02]. A significant discrepancy was also found in the inclusion of references from local experts, which was far more common in US-based searches [median (IQR), 2 (1-2) vs 5 (4-5); p<0.0001]. Although all raters found the references to be appropriate for the cases [median (IQR), 2 (1-2) vs 2 (1-2); p=0.42], both groups were neutral regarding the concern for patient harm if OE were used by an inexperienced provider [median (IQR) 3 (2-4) vs 3 (2-4); p=0.52].

We analyzed results from additional questions on a Likert scale (1= Strongly Agree and 5= Strongly Disagree) (Table 4) comparing US-based search results vs the global group. Notably, both groups had an identical median score of two; the significant p-value reflects the wider interquartile range in the global searches, indicating greater disagreement among global raters rather than a consensus that differentials were less relevant or pertinent. Analysis of search results showed most references were based in North America, lacking local authoritative guidelines for searches conducted in various global locations, such as a lack of referencing Canadian Cardiovascular Society guidelines for a case of acute coronary syndrome and searches in Global South countries retrieving older evidence. The differentials in Global South searches lacked contextualization of infectious disease causes, lacking mention of malaria and TB for searches in Kenya. Accessibility was variable across different sites, with Global South countries having numerous challenges and India lacking access. The quality of management plans varied drastically between searches in different countries with US-based searches providing detailed management plans and searches in global locations yielding very basic search results.

## Discussion

Principal findings

In our study using the RAS, OE responses demonstrated higher ratings of accuracy, efficiency, and coherence (87.5%- 89%) with lower scores for comprehensiveness, argument and evidence integration, creativity, and originality (65%- 75%). Comparative analysis showed statistically significant differences between US-based search results and the searches in global locations across several domains. The global searches demonstrated greater variability in differential diagnosis (p=0.03), reflecting wider disagreement among non-US raters rather than a consensus of lower relevance, as both groups had an identical median of two. US-based clinicians rated management plans as more aligned with published guidelines and reported greater perceived value, with inclusion of locally authored references. These exploratory findings suggest that while OE performs well on foundational RAS quality indicators, measurable variability exists in contextual adaptation and guideline alignment with geographic settings.

Overall, the references cited in the 80 searches showed sources from the Global North, lacking Global South references. Our findings are consistent with a well-documented issue in clinical research and machine learning: the disproportionate overrepresentation of US and high-income country datasets [4]. This skewness leads to algorithms that learn patterns not reflective of worldwide disease incidence, comorbidity profiles, or health-system constraints [24]. The underrepresentation is further compounded by systemic barriers faced by researchers in low- and middle-income countries (LMICs), including limited access to resources and institutional barriers to open-access publishing, which perpetuates the cycle of data inequality [25]. While OE aims to democratize access to information, its reliance on freely available abstracts rather than full-text articles only partially addresses this core accessibility issue [26]. In addition, publication bias, which favors positive outcomes, can lead to overstated findings and means that AI systems trained on existing literature may propagate an unbalanced and incomplete view of effective care [27,28].

Our findings should be contextualized against the recent large-scale evaluations of OE that reached opposing conclusions within US settings [21,22]. The divergence in their conclusions likely reflects differences in query origin institutional generative pre-trained transformer (GPT) versus native OE queries, evaluator sample size and specialty matching, and evaluation design. Notably, OE was queried through browser interfaces rather than via an application programming interface (API), which may have introduced formatting and response differences not attributable to the underlying model itself. Our exploratory study does not resolve the debate but contributes a dimension absent from both evaluations. Both evaluations queried OE from US settings and used US-based evaluators and were not designed to test its application in global contexts. Our finding that OE performs well on core features such as accuracy and efficiency is consistent with Feng et al.'s study while demonstrating geographic variability when queries originate in non-US settings, highlighting the fact that the existing evaluations describe OE’s performance within its primary deployment context rather than its global performance. The absence of Global South references across all 10 cases from six different countries, with citation guidelines that were dated in Global South countries, and the absence of suggestion of malaria for a fever differential for searches conducted in Kenya are findings that neither of the current studies nor evaluations were designed to detect. These findings are hypothesis-generating and require replication in larger controlled studies, but they identify a geographic dimension for applicability that is consequential for clinical deployment and absent from current benchmarks.

In addition, several authors reported challenges with accessibility; although accessibility was not formally measured, these challenges highlight practical implementation barriers that may affect global usability. Equitable deployment of these AI solutions not only requires algorithmic performance consistency but also reliable accessibility with variable internet and bandwidth constraints. In its current form, OE may exacerbate the gap between Global North and South countries with accessibility issues and variability in the quality of outputs that lack contextualization. Ensuring proper safeguards around access is essential to promote responsible use and minimize potential risks. The barriers to access may have introduced a selection bias since evaluations could only be conducted on outputs that were successfully retrieved.

Implications and recommendations for clinical practice

Health professionals worldwide must be aware of geographic output variability when using applications like OE [29] and critically evaluate the real-world applicability of management recommendations. While existing evaluations have assessed OE’s performance with frontier models, geographic variability and evaluations of output quality and contextualization of management needs to be studied further. Based on our preliminary findings, we recommend that OE search outputs be used as a preliminary tool for exploring clinical questions, rather than a definitive guide for decision-making. Clinicians must critically appraise the quality and applicability of the output, paying close attention to comprehensiveness and contextual relevance. To avoid AI dependency, it is crucial to remain aware of the variability in LLM outputs.

To inform future development, our study underscores target areas. Retrieval should be weighted by disease burden as well as literature density so epidemiologically dominant but under-published conditions are not systematically omitted. Geographic context provided in a query should propagate evidence-based results that are locally relevant rather than merely altering the prose for the response. Where localization is not possible, the tool should acknowledge the limitation, rather than assert generalized management. Model limitations regarding regional applicability should be acknowledged transparently [30].

Limitations

This study has several limitations. Though we used a validated assessment tool, there will be an inherent subjective judgment introducing evaluator variability. Some evaluator variability is unavoidable, because clinicians evaluated responses pertinent to their own region and specialty, as these clinicians were best positioned to judge local clinical appropriateness. These rater effects cannot be statistically separated from regional differences. RAS scores should therefore be interpreted as appraisals by clinicians within their own practice contexts. Future studies could explore some of the additional factors. Also, being an exploratory study, not every reviewer assessed every case. Our study was underpowered to provide reliable estimates of inter-rater reliability. We utilized a small convenience sample with narrow global representation; given the acknowledged underpowering, the reported p-values must be interpreted as descriptive and hypothesis-generating rather than confirmatory statistically. Due to unequal group cohorts (21 vs. 59), the ratings should be interpreted cautiously. Additionally, four out of eight clinicians were from Taiwan, which further limits the representativeness of global findings. The additional RAS questions developed for this study were not formally validated beyond face validity through team consensus. We collected results through the browser interface rather than the API. We are aware that OE is updated continuously, and our findings measure the results generated during the study period. We acknowledge that the same searches may yield different results if performed currently due to model refinement, but the results inform variability in outputs that clinicians need to be aware of. We did not run repeated queries within the same country to measure stochastic variability.

The Wilcoxon rank-sum test assumes independent observations; because individual clinicians contributed multiple ratings, this assumption was not fully met, and analyses did not account for clustering by rater. In addition, pooling ratings from five countries into a single non-US group may obscure differences between searches conducted in different locations. With one to four raters per country, comparisons reflect individual clinicians' appraisals rather than country-level estimates.

## Conclusions

In this exploratory study, OE demonstrated variability in output for clinical applications across geographic contexts with US based searches showing greater alignment with guidelines and locally authored references compared to non-US searches. Clinicians should critically appraise AI outputs prior to application in real-world contexts, modeling responsible use. As AI solutions become more integrated into healthcare, developers and health systems must ensure these tools are built with fairness, equity, and transparency as core requirements. Future efforts should include large-scale studies with validated cross-cultural instruments, structured algorithmic bias auditing, and transparent reporting of training data to support global deployment of these solutions.

## Appendices

Appendix 1 

**Table 5:** CHART checklist CHART: Chatbot Assessment Reporting Tool.

CHART Checklist			
Heading	No	Chart checklist item	Page No
Title and abstract			
Title	1a	State that the study is assessing one or more generative AI-driven chatbots for clinical evidence or health advice.	
Abstract/summary	1b	Apply a structured format, if applicable.	
Introduction			
Background	2a	State the scientific background, rationale, and healthcare context for evaluating the generative AI-driven chatbot(s), referencing relevant literature when applicable.	
	2b	State the aims and research questions including the target audience, intervention, comparator(s), and outcome(s).	
Methods			
Model identifiers	3a	State the name and version identifier(s) of the generative AI model(s) and chatbot(s) under evaluation, as well as their date of release or last update.	
	3b	State whether the generative AI model(s) and chatbot(s) are open-source or closed-source/proprietary.	
Model details	4a	State whether the generative AI model was a base model or a novel base model, tuned model, or fine-tuned model.	
	4b	If a base model is used, cite its development in sufficient detail to identify the model.	
	4c	If a novel base model, tuned model, or fine-tuned model is used, describe the pre- and/or post-implementation/deployment data and parameters.	
Prompt engineering	5a	Describe the evolution of study prompt development.	
	5ai	Describe the sources of prompts.	
	5aii	State the number and characteristics of the individual(s) involved in prompt engineering.	
	5aiii	Provide details of any patient and public involvement during prompt engineering.	
	5b	Provide study prompts.	
Query strategy	6a	State route of access to generative AI model.	
	6b	State the date(s) and location(s) of queries for the generative AI-driven chatbot(s) including the day, month, and year as well as city and country.	
	6c	Describe whether prompts were input into separate chat session(s).	
	6d	Provide all generative AI-driven chatbot output/responses	
Performance evaluation	7a	Define the ground truth or reference standard used to define successful generative AI-driven chatbot performance.	
	7b	Describe the process undertaken for generative AI-driven chatbot performance evaluation.	
	7bi	State the number and characteristics of team members involved in performance evaluation.	
	7bii	Provide details of any patients and public involvement during the evaluation process.	
	7biii	State whether evaluators were blinded to the identity of the generative AI-driven chatbot(s) under assessment.	
Sample size	8	Report how the sample size was determined.	
Data analysis	9a	Describe statistical analysis methods, including any evaluation of reproducibility of generative AI-driven chatbot responses.	
	9ai	Report the measures used for performance evaluation.	
Results			
	10a	Report the performance evaluation undertaken including the alignment between generative AI-driven chatbot output and ground truth or reference standard using quantitative or mixed methods approaches as applicable.	
	10b	For responses deviating from the ground truth or reference standard, state the nature of the difference(s).	
	10c	Report the evaluation for potentially harmful, biased, or misleading responses.	
Discussion			
	11a	Interpret study findings in the context of relevant evidence.	
	11b	Describe the strengths and limitations of the study.	
	11c	Describe the potential implications for practice, education, policy, regulation, and research.	
Open science			
Disclosures	12a	Report any relevant conflicts of interest for all authors.	
Funding	12b	Report sources of funding and their role in the conduct and reporting of the study.	
Ethics	12c	Describe the process undertaken for ethical approval.	
	12 ci	Describe the measures taken to safeguard data privacy of patient health information, as applicable.	
	12cii	State whether permission/licensing was obtained for the use of original, copyrighted data.	
Protocol	12d	Provide a study protocol.	
Data availability	12e	State where study data, code repository, and model parameters can be accessed.	

Appendix 2 

**Table 6:** Hypothetical cases Hypothetical natural language query entered by authors in their area of specialty.

I am a clinician working in x hospital in X state, country.		
Case 1 Describe how to manage an 8-year-old child presenting with sudden onset of high fever, severe headache, retro-orbital pain, joint and muscle pains, and petechial rash.		
Case 2 Guide me on how to manage a 70 yo male with chest pain, he has Type2 DM, Hypertension, Hypercholesterolemia and presents with acute onset chest pain radiating to his L arm		
Case 3 Walk me through how to manage a 29-year-old woman presents with a chronic cough lasting more than 4 weeks, mild hemoptysis, significant weight loss, night sweats, and fatigue. Chest radiograph reveals apical infiltrates and cavities.		
Case 4 Suggest management of a 10-year-old with sudden onset of sore throat, fever, cervical lymphadenopathy, tonsillar exudates, and no significant cough.		
Case 5 Provide a treatment plan for a 56-year-old man presents for a routine checkup. Blood pressure readings consistently measure above 150/95 mmHg, but he reports no symptoms. Fundoscopy shows mild hypertensive retinopathy.		
Case 6 Discuss differentials of a 16-year-old adolescent presents with shortness of breath, audible wheezing, chest tightness, and a dry cough following exposure to dust and pollen. Symptoms partially relieved by bronchodilators.		
Case 7 What are the treatment consideration for a 68-year-old man with a 40-year history of smoking presents with chronic productive cough, progressive exertional dyspnea, wheezing, and frequent respiratory infections.		
Case 8 Describe how to manage a 75-year-old adult with abrupt onset fever, productive cough with rusty-colored sputum, shortness of breath, pleuritic chest pain, and possible confusion.		
Case 9 What are the management considerations for a 16-year-old adolescent with persistent sore throat, fever, severe fatigue, posterior cervical lymphadenopathy, and hepatosplenomegaly.		
Case 10 Describe differentials I need to consider when managing a 10-month-old infant with acute watery diarrhea, vomiting, fever, and signs of dehydration.		

Appendix 3 

All prompts and their corresponding responses from each location can be accessed at https://bit.ly/OESEARCH.
